# Antifungal activity of extracts from Atacama Desert fungi against *Paracoccidioides brasiliensis* and identification of *Aspergillus felis* as a promising source of natural bioactive compounds

**DOI:** 10.1590/0074-02760150451

**Published:** 2016-03

**Authors:** Graziele Mendes, Vívian N Gonçalves, Elaine M Souza-Fagundes, Markus Kohlhoff, Carlos A Rosa, Carlos L Zani, Betania B Cota, Luiz H Rosa, Susana Johann

**Affiliations:** 1 Universidade Federal de Minas Gerais, Instituto de Ciências Biológicas, Departamento de Microbiologia, Belo Horizonte, MG, Brasil; 2 Fundação Oswaldo Cruz, Centro de Pesquisa René Rachou, Laboratório de Química de Produtos Naturais, Belo Horizonte, MG, Brasil; 3 Universidade Federal de Minas Gerais, Departamento de Fisiologia e Biofísica, Belo Horizonte, MG, Brasil

**Keywords:** rock-inhabiting fungi, Atacama Desert, Paracoccidioides brasiliensis, antifungal, Aspergillus felis

## Abstract

Fungi of the genus *Paracoccidioides* are responsible for paracoccidioidomycosis. The occurrence of drug toxicity and relapse in this disease justify the development of new antifungal agents. Compounds extracted from fungal extract have showing antifungal activity. Extracts of 78 fungi isolated from rocks of the Atacama Desert were tested in a microdilution assay against *Paracoccidioides brasiliensis Pb*18. Approximately 18% (5) of the extracts showed minimum inhibitory concentration (MIC) values*≤* 125.0 µg/mL. Among these, extract from the fungus UFMGCB 8030 demonstrated the best results, with an MIC of 15.6 µg/mL. This isolate was identified as *Aspergillus felis* (by macro and micromorphologies, and internal transcribed spacer, β-tubulin, and ribosomal polymerase II gene analyses) and was grown in five different culture media and extracted with various solvents to optimise its antifungal activity. Potato dextrose agar culture and dichloromethane extraction resulted in an MIC of 1.9 µg/mL against *P. brasiliensis* and did not show cytotoxicity at the concentrations tested in normal mammalian cell (Vero). This extract was subjected to bioassay-guided fractionation using analytical C18RP-high-performance liquid chromatography (HPLC) and an antifungal assay using *P. brasiliensis*. Analysis of the active fractions by HPLC-high resolution mass spectrometry allowed us to identify the antifungal agents present in the *A. felis* extracts cytochalasins. These results reveal the potential of *A. felis* as a producer of bioactive compounds with antifungal activity.

Paracoccidioidomycosis (PCM) is a human systemic mycosis endemic in Latin America (Tavares et al. 2005). Approximately 10 million people in this region are infected (Stürme et al. 2011), with 85% of cases occurring in Brazil (Andrade et al. 2005), and specific social groups, such as rural workers, being particularly affected (Shikanai-Yasuda et al. 2006). PCM is acquired by inhaling airborne propagules derived from the mycelial form of *Paracoccidioides brasiliensis* (Tavares et al. 2005) and *Paracoccidioides lutzii*(Teixeira et al. 2009). They adhere to the alveolar epithelium, where they transform into pathogenic yeasts (Torres et al. 2010).

Despite the effectiveness of treatments with currently available drugs (amphotericin B, azoles, and sulfonamides), they require long term administration protocols capable of causing toxic effects (Borges-Walmsley et al. 2002, Palmeiro et al. 2005, Shikanai-Yasuda et al. 2006, Visbal et al. 2011).

In addition, antifungal chemotherapy does not ensure the complete elimination of the fungus from the patient (Travassos & Taborda 2012). The discovery of new antifungal agents with higher efficacies and fewer side effects is needed in order to increase treatment options for this infection.

Abadio et al. (2015) using the rational combination of molecular modelling simulations and virtual screening identified compounds against thioredoxin reductase of *P. lutzii*, which is a promising target for drugs. Transcriptome is another potential experimental strategy to elucidate the mechanism of action of bioactive compounds using the change in gene expression. Argentilactone, for example, appears to be capable of modulating cellular targets by inducing oxidative stress and interfere with cell wall biosynthesis in*P. lutzii* (Araújo et al. 2016). Proteomic profile of this fungus indicated a global metabolic adaptation in the presence of argentilactone. Enzymes of important pathways were repressed in*P. lutzii*, while proteins involved in cell rescue, defense, and stress response were induced in the presence of argentilactone (Prado et al. 2015).

Rock-inhabiting fungi are among the most stress-tolerant organisms on Earth, able to cope with the variety of stressors associated with bare rocks in environments of hot and cold extremes (Tesei et al. 2012). These surfaces are unique habitats where rapid changes in radiation, temperature, water and nutrient availability represent a challenge to microbial survival in different environments across the world (Gueidan et al*.* 2008).

The Atacama Desert may be the oldest desert on Earth (Azua-Bustos et al. 2012). Atacama’s long-standing aridity adds value to the study of biological adaptations, since that, organisms have been exposed to challenging environmental conditions for sufficiently long to bear witness to evolution and natural selection processes (Wierzchos et al. 2013). It is believed that species adapted to live in such environments constitute potential sources of enzymes with special characteristics and novel genes with possible industrial applications (Dalmaso et al. 2015).

The present study aimed to evaluate the activity of crude extracts from a collection of fungi isolated from the Atacama Desert against the human pathogenic fungus *P. brasiliensis*. Extract of the strain UFMGCB 8030 showed outstanding antifungal activity against this fungus of medical importance, and thus it was selected for further investigation.

## MATERIALS AND METHODS

*Fungal material* - The 78 fungal isolates used in this study were obtained from rocks collected in the Atacama Desert (Gonçalves et al. 2015). These fungi have been deposited in the Collection of Microorganisms and Cells of the Federal University of Minas Gerais (UFMG), Brazil, under codes UFMGCB 8010-8090 (Table I).

**TABLE I:** Minimum inhibitory concentrations (MIC) of extracts of fungi isolated from Atacama Desert rocks against *Paracoccidioides brasiliensis Pb*18

Fungal species	UFMGCB^*a*^	MIC (µg/mL)
*Alternaria* cf.*arborescens*	8010	500.0
*Aspergillus felis*	8011	**250.0**
*Alternaria sp. 1*	8012	500.0
*Alternaria sp. 2*	8013	500.0
*Cladosporium halotolerans*	8014	-
*Neosartorya* cf. *udagawae*	8015	**125.0**
*Cladosporium* cf.*cladosporioides*	8017	500.0
*A.* cf*. arborescens*	8018	-
*A. felis*	8019	500.0
*Hypoxylon* cf. *trugodes*	8020	500.0
*N.* cf*. udagawae*	8021	**62.5**
*Fusarium oxysporum*	8023	-
*A. felis*	8024	**31.2**
*A. felis*	8025	**-**
*A. felis*	8026	**31.2**
*Eupenicillium javanicum*	8027	500.0
*Cladosporium* cf.*oxysporum*	8028	-
*Aspergillus sp.*	8029	-
*A. felis*	8030	**15.6**
*Aspergillus lentulus*	8031	500.0
*Neosartorya sp. 2*	8032	500.0
*F. oxysporum*	8033	500.0
*E. javanicum*	8034	500.0
*A. lentulus*	8035	-
*Penicillium* cf.*puvillorum*	8036	-
*Neosartorya sp. 2*	8037	500.0
*E. javanicum*	8038	-
*Neosartorya sp. 2*	8039	500.0
*A. felis*	8040	-
*C. halotolerans*	8041	-
*C. halotolerans*	8042	-
*Penicillium crysogenum*	8043	500.0
*Didymellaceae sp.*	8044	-
*P. crysogenum*	8045	500.0
*Aspergillus persii*	8046	-
*Aspergillus westerdijkiae*	8047	500.0
*Cladosporium* cf.*gossypiicola*	8048	-
*P. crysogenum*	8049	500.0
*Macroventuria* cf.*anomachaeta*	8050	-
*Penicillium* cf*. citrinum*	8051	-
*P.* cf*. citrinum*	8052	-
*P. crysogenum*	8053	-
*P. crysogenum*	8054	-
*P. crysogenum*	8055	500.0
*C. halotolerans*	8056	-
*P. crysogenum*	8057	500.0
*Aspergillus sydowii*	8058	-
*P.* cf*. citrinum*	8059	-
*P.* cf*. citrinum*	8060	-
*Devriesia sp.*	8061	-
*P.* cf*. citrinum*	8062	-
*Neosartorya sp. 2*	8063	-
*Neosartorya sp. 2*	8064	-
*Neosartorya sp. 2*	8065	-
*Neosartorya sp. 1*	8066	-
*Neosartorya sp. 1*	8067	-
*Neosartorya sp. 2*	8068	-
*Neosartorya sp. 2*	8069	-
*C. halotolerans*	8070	-
*Neosartorya sp. 2*	8071	-
*Neosartorya sp. 2*	8072	-
*P.* cf*. citrinum*	8073	-
*P. crysogenum*	8074	500.0
*C. halotolerans*	8075	-
*C.* cf. *gossypiicola*	8076	-
*C. halotolerans*	8077	-
*P.* cf*. citrinum*	8078	-
*Neosartorya* cf*. udagave*	8079	-
*P.* cf*. citrinum*	8080	-
*P. crysogenum*	8081	-
*Pseudogymnoascus* cf.	8082	-
*Cladosporium* cf*.*	8083	-
*Cladosporium* cf*.*	8084	-
*Cladosporium* cf*.*	8085	-
*A.* cf*. arborescens*	8086	-
*C. halotolerans*	8087	-
*P.* cf*. citrinum*	8089	500.0
*P.* cf*. citrinum*	8090	-

*a*: Collection of Microorganisms and Cells of the Federal University of Minas Gerais, Brazil; -: no antifungal activity. Bold values: good antifungal activity.

*Fungal cultivation and preparation of extracts for biological assays*- All fungal isolates were cultivated and extracts prepared according to protocols established by Rosa et al. (2013). A stock solution of each extract was prepared in dimethyl sulfoxide (DMSO) (Merck, USA) at a concentration of 100 mg/mL and stored at -20ºC. Extract of sterile yeast mold medium (YM) (0.3% yeast extract, 0.3% malt extract, 0.5% peptone, 2% glucose, and 2% agar), generated using the same extraction protocol, was used as a control in the screening procedure.

*Antifungal assay* - *Fungal isolate and inoculum* - Antifungal activity of the extracts was evaluated using *P. brasiliensis Pb*18 (Fungi Collection of the Faculty of Medicine of São Paulo University, Brazil). Isolate *Pb*18 belongs to the cryptic phylogenetic species S1 (Matute et al. 2006) and was maintained at the Microbiology Department of the UFMG by weekly transfer onto solid yeast peptone dextrose medium (1% yeast extract, 0.1% peptone, 1% dextrose, and 2% agar) at 37ºC. Isolated *Pb*18 cells were suspended in sterile saline and the transmittance of the resulting suspension at a wavelength of 530 nm was adjusted to 70% (1-5 × 10^6^ cells/mL) using a spectrophotometer (SP-22; Biospectro, Brazil). The yeast-cell stock suspension was diluted in a 1:10 solution of RPMI-1640 medium (Sigma-Aldrich, USA) plus 3-(*N*-morpholino)-propanesulfonic acid broth (Sigma-Aldrich) for a final inoculum of 1-5 × 10^5^ cells/mL (Cruz et al. 2012).

*Antifungal activity screen* - Extracts were diluted in RPMI medium for final concentrations of 500 µg/mL with DMSO at 0.5% v/v. RPMI medium with inoculum was used as a growth control, while the former was used on its own as a sterility control. DMSO (0.5% v/v) was used as a control for toxicity and itraconazole (0.05-0.0005 µg/mL) (Sigma-Aldrich) as a susceptibility control. The 96-well plates were prepared in duplicate and incubated at 37ºC for 10 days. After this period, the plates were visually assessed and 10 µL of 5 mg/mL thiazolyl blue tetrazolium bromide (MTT) (Sigma-Aldrich) was added to each well prior to 4-h incubation. Following MTT metabolism, 100 µl of 5% v/v sodium dodecyl sulfate/isopropanol was added per well. The absorbance of test wells was measured at 530 nm using a microtitre plate spectrophotometer (VersaMax; Molecular Devices, USA) and compared with that of the growth control well. The inhibition of yeast growth (% inhib.) was calculated as a percentage according to the following equation where OD signifies optical density:


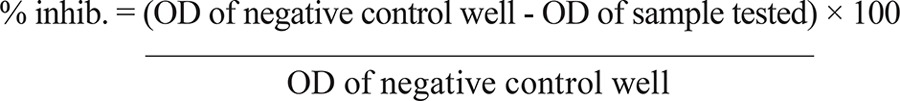


Extracts demonstrating 70% inhibition of isolate *Pb*18 growth were considered active and subjected to a minimum inhibitory concentration (MIC) assay.

*Determination of MIC* - Microdilution assays were performed using the same conditions as those described for the antifungal activity screen (CLSI 2008, Johann et al. 2010). By dilution in RPMI-1640 broth, 10 two-fold serial dilutions of the selected extracts, ranging from 500.0-0.9 µg/mL, were tested. DMSO (0.5% v/v) was used as a control for toxicity and itraconazole (0.05-0.0005 µg/mL) as a susceptibility control. The MIC was considered to be the lowest concentration completely inhibiting *Pb*18 growth compared to the growth control, expressed in µg/mL. All tests were performed in duplicate in three independent experiments.

*Molecular identification* - The DNA extraction protocol and amplification of the internal transcribed spacer (ITS) region, achieved using the universal primers ITS1 and ITS4 (White et al. 1990), have been described by Rosa et al. (2009). Amplification of β-tubulin (Glass & Donaldson 1995) and ribosomal polymerase II genes (RPB2) (Houbraken et al*.* 2012) was performed with Bt2a/Bt2b and RPB2-5F-Pc/RPB2-7CR-Pc 7CR primers, respectively, according to protocols established by Godinho et al. (2013). To achieve species-rank identification based on ITS, β-tubulin, and RPB2 data, consensus sequences were aligned using all sequences of related species retrieved from the National Center for Biotechnology Information GenBank database using the Basic Local Alignment Search Tool (Altschul et al. 1997). The sequences obtained were subjected to ITS, β-tubulin, and RPB2-based phylogenetic analyses using comparisons with sequences of type species deposited in GenBank, with estimations calculated by MEGA v.5.0 (Tamura et al. 2011). The maximum composite likelihood method was employed to estimate evolutionary distances, with bootstrap values calculated from 1,000 replicate runs. Information concerning fungal classification generally follows Kirk et al. (2008) and the MycoBank (mycobank.org) and Index Fungorum (indexfungorum.org) databases.

*Morphological identification* - Macroscopic fungal parameters (colony colour and texture, border type, and radial growth rate) and colony diameters were observed on Czapek yeast autolysate (CYA) (0.5% w/v yeast extract, 3.5% w/v Czapeck, 2% w/v agar) and malt extract agar (MEA) [2% w/v malt extract, 0.1% w/v peptone, 2% w/v glucose (HiMedia, India)]. Three-point inoculations of the fungus UFMGCB 8030 were incubated for seven days in the dark at 25ºC. Fungal reproductive structures were produced by microculture technique, stained with lactophenol cotton blue (0.05% w/v), and evaluated under an optical microscope (DM750; Leica, Germany) at 40X magnification (Klich 2002).

*Cultivation and extraction of UFMGCB 8030 using different culture media* - The fungus UFMGCB 8030 was grown on the following five culture media in order to evaluate the antifungal activity of its extracts: potato dextrose (PDA) [2% w/v glucose, 30% w/v potato infusion (HiMedia)], YM, MEA, corn meal (HiMedia), and minimal medium containing 6.98 g/L K_2_HPO_4_, 5.44 g/L KH_2_PO, and 4.1 g/L (NH_4_)_2_SO_4_, and supplemented with 5, 10, 15, 20, and 30 g/L glucose. The cultures were incubated at 25 ± 2ºC for 15 days and extracted with ethanol (Vetec, Brazil) for 24 h at ambient temperature. After filtration, the organic phase was concentrated on a rotary evaporator. Residual solvent was removed with a SpeedVac system (Savant SPD 121P; Thermo Scientific, USA) at 40ºC to yield crude extracts.

*Production of UFMGCB 8030 extracts using different solvents* - UFMGCB 8030 was grown on PDA medium at 25 ± 2ºC for 15 days, with cultures being subjected to extraction three times at 48 h intervals using 20 mL of hexane, dichloromethane (DCM), ethyl acetate or ethanol (all Vetec). The extracts were obtained by the procedure described above.

*Cytotoxicity assay* - The VERO (African green monkey kidney cells) lineage was used as a model of normal cells. This lineage was maintained in the logarithmic phase of growth in Dulbecco’s modified Eagle’s medium supplemented with 100 IU/mL penicillin and 100.0 μg/mL streptomycin enriched with 5% foetal bovine serum. VERO cells were maintained at 37ºC in a humidified incubator with 5% CO_2_ and 95% air. The medium was changed twice weekly and the cells were regularly examined and used until 20 passages. Vero cells were seeded at a density of 1 × 10^4^ cells before being pre-incubated for 24 h at 37ºC to allow for their adaptation prior to addition of the test sample. The extract was dissolved in DMSO (0.5% v/v) before dilution and tested over a range of concentrations (8 nonserial dilutions from 100-1.5 µg/mL). All cell cultures were incubated in a humidified 5% CO_2_/95% air atmosphere at 37ºC for 48 h. The negative control comprised treatment with 0.5% v/v DMSO. Controls included drug-containing medium (background) and drug-free complete medium. Drug-free complete medium was used as a control (blank) and was treated in the same way as the drug-containing media. Results were expressed as a percentage of inhibition of cell viability compared to the 0.5% DMSO control and were calculated as follows: % inhibition of cell viability (%) = 100 - (mean OD treated - mean OD background)/(mean OD untreated culture, i.e., 0.5% DMSO - mean OD blank wells) x 100. Interactions between compounds and media were estimated on the basis of variations between drug-containing media and drug-free media to avoid false-positives or false-negatives (Monks et al. 1991). All samples were tested in triplicate in two independent experiments.

*Chromatographic separation of UFMGCB 8030 DCM extract and identification of active compounds* - Analytical chromatography was performed on a reversed-phase high-performance liquid chromatography (RP-HPLC) system (Shimadzu, Japan) equipped with a manual injector, two pumps (LC-10A), and a diode array detector (SPD-M10A). DCM extract (500 µg) was injected into an analytical HPLC column [Shim-pack ODS, 4 µm, 3.9 × 150 mm (Shimadzu)] and eluted at a flow rate of 1 mL/min using a gradient of 15-100% acetonitrile (ACN) in water for 16 min, followed by 100% ACN for 8 min. The effluent was collected in a 96-well plate (300 µL per well in 80 wells) using a fraction collector (SF2120; Advantec MFS, USA). The experiment was repeated four times and the plates obtained were dried in a SpeedVac vacuum centrifuge at 40ºC. Fractions from two plates were dissolved in 100 µL RPMI medium containing 0.5% v/v DMSO before being transferred to fresh plates for the*P. brasi- liensis Pb*18 bioassay. Fractions showing 70% inhibition of isolate *Pb*18 growth were considered active.

Active compounds were dissolved by addition of ACN to the appropriate wells prior to liquid chromatography-mass spectrometry (LC-MS) [tandem MS (MS/MS)] analysis. This was performed on a Nexera UHPLC system (Shimadzu) coupled to a maXis ETD high-resolution ESI-QTOF mass spectrometer (Bruker, USA) and controlled by the Compass 1.5 software package (Bruker). Fractions (20 µL) were injected into a Shim-Pack XR-ODS III column [C18, 2.2 µm, 2.2 × 200 mm (Shimadzu)] at 40ºC using a flow rate of 200 µL/min. The components of the mobile phase, A and B (0.1% formic acid in water and ACN, respectively), formed an eluent gradient as follows: 5% B for the initial 0.5 min, then a linear gradient to 100% B over 12.5 min, and a final hold for 1 min of 100% B. Ultraviolet chromatograms were recorded at wavelengths of 214 and 254 nm. The mass spectra were acquired in positive mode at a spectra rate of 2 Hz. Ion-source parameters were set to 500 V end plate offset, 4,500 V capillary voltage, 2.0 bar nebuliser pressure, and 8.0 L/min and 200ºC dry gas flow and temperature, respectively. Data-dependent of precursor fragmentation was performed at collision energies of 40 eV. Ion cooler settings were optimised within an*m*/*z* range of 40-1,000 using a solution of 10 mM sodium formate in 50% 2-propanol for calibration. Mass calibration was achieved by an initial ion-source infusion of 20 µL calibration solution and post-acquisition recalibration of the raw data.

Compound detection was performed by chromatographic peak analysis with subsequent formula determination according to exact mass and isotope pattern (MS1) and database comparison of compound fragment spectra (MS2). An in-house database of standard compounds and the public spectra database MassBank (Horai et al. 2010) served as sources of reference ESI fragment spectra.

LC-MS mass data files were used to identify the active compounds from KNApSAcK and SciFinder/Chemical Abstracts Service databases. Manual interpretation of MS/MS spectra was also performed using the MassBank database.

## RESULTS

When the 78 extracts of fungi obtained from rocks in the Atacama Desert were tested at a single concentration (500.0 µg/mL) against *P. brasiliensisPb*18 35% were found to inhibit 70% of growth. These were considered to be active and their MICs were determined. Approximately 18% (5) of these active extracts exhibited MICs ≤ 125.0 µg/mL (Table I). UFMGCB 8030 extract demonstrated the lowest MIC with a value of 15.6 µg/mL followed by those of isolates UFMGCB 8024 and UFMGCB 8026, with values of 31.2 µg/mL, and UFMGCB 8021, with an MIC of 62.5µg/mL. The fungal isolates have been identified based on ITS sequence analysis by Gonçalves et al. (2015), being grouped into 30 species belonging to 13 genera. The fungi providing the most active extracts in the present work were *Neosartorya* cf.*udagawae* (UFMGCB 8015 and 8021, with MICs of 125.0 and 62.5 µg/mL, respectively) and *Aspergillus felis* (UFMGCB 8024, 8026, and 8030, with MICs of 31.2-15.6 µg/mL) (Table I)*.* Among the most active extracts, *A. felis* UFMGCB8030 was of particular interest, showing promising activity against *P. brasiliensis Pb*18 (MIC = 15.6 µg/mL). Thus, a more detailed study to identify both this fungus and the active compounds in its extract was performed.

**Fig. 1:**
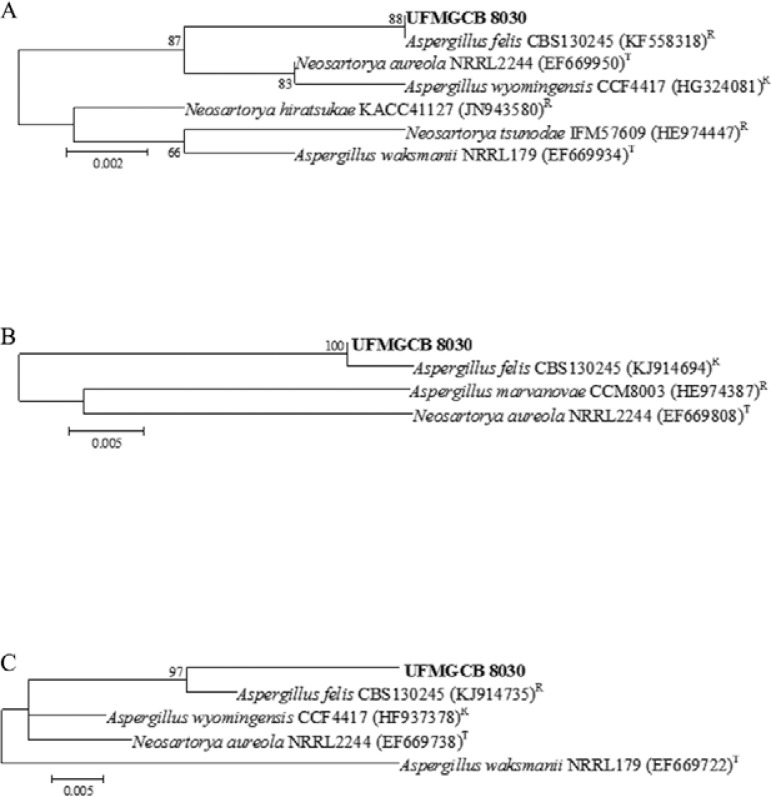
: phylogenetic analysis of nucleotide sequences obtained from fungus UFMGCB 8030 (in bold) associated with rocks from the Atacama Desert in comparison with type (T) and reference (R) sequences deposited in GenBank. Trees were constructed based on ITS1-5.8S-ITS2 (A), β-tubulin (B), and ribosomal polymerase II gene (C) sequences using the maximum composite likelihood model.

**Fig. 2:**
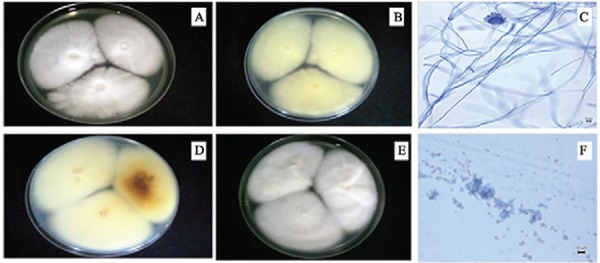
: *Aspergillus felis* colonies after seven days at 25°C on malt extract agar (A, B) and Czapeck yeast autolysate agar (D, E). Conidiophores and conidia (C, F) at 40X magnification (10 µm). Top-down (A, E) and reverse (B-D) aspects of cultures.

Although the ITS-based identification of *A. felis* UFMGCB 8030 gave satisfactory results (Gonçalves et al. 2015), we also sequenced its β-tubulin and RPB2 (Fig. 1). A combination of phylogenetic evaluation (Fig. 1) and analysis of micro and macro-morphological features (Fig. 2) increased the degree of confidence in this identification.

The ITS nucleotide sequence showed 100% query coverage and 100% similarity with that of *A. felis* (GenBank accession KF558318). In addition, the β-tubulin and RPB2 sequences of this isolate shared 84% and 100% query coverage and 99% and 98% of similarity, respectively, with the corresponding *A. felis* sequences (GenBank accessions KJ914694 and KJ914735, respectively). ITS, β-tubulin, and RPB2 references or type species sequences were retrieved from GenBank and used in a neighbour-joining phylogenetic analysis with 1,000 bootstrap replicates (Fig. 1). This approach revealed distinct clustering of the organism of interest in this study with*A. felis*, confirming it to be the species most genetically similar to isolate UFMGCB 8030.

The following characteristics of the *Aspergillus* isolate were observed, as shown in Fig. 2: colony diameters of 5.0 and 5.5 cm after seven days at 25ºC on CYA and MEA media, respectively, and sporulation on MEA at 25ºC on the 14th day of culture. On CYA medium, colony texture is mostly floccose; colonies are usually white, with a cream-to-light-brown reverse, and often sporulate poorly. Furthermore, yellow soluble pigments are diffused into the agar. On MEA, colonies are somewhat velvety with greenish sporulation occurring after seven days. Colonies have a cream reverse. Conidiophores are uniseriate with greenish stipes (12 × 5.0 µm) and green globose conidia 1.5-2.5 µm in length. Phialides are 6.0 × 2.0 µm and vesicles are pyriform with a diameter of 13 mm. After taxonomic analysis using molecular and morphological methods, fungus UFMGCB 8030 was confirmed to be *A. felis* (Barrs et al. 2013).

In the present study, the production of bioactive compounds was assessed by varying certain culture conditions of *A. felis* UFMGCB 8030 and testing the resulting extracts with a *P. brasiliensis* bioassay. In regard to culture media, the most striking results were obtained with extracts from fungi cultivated on PDA (MIC = 7.8 µg/mL) followed by those from YM and corn meal cultures (MIC = 15.6 µg/mL). On MEA medium, the MIC of the ethanol extract was 62.5 µg/mL (Table II). Extracts obtained after cultivation of this isolate on minimal medium supplemented with glucose showed no antifungal activity against *Pb*18.

**TABLE II:** Minimum inhibitory concentrations (MIC) against*Paracoccidioides brasiliensisPb*18 of ethanol extracts from *Aspergillus felis* (UFMGCB 8030) grown on different culture media

Culture medium (g/L)	MIC (µg/mL)
MM (5)	-
MM (10)	-
MM (15)	-
MM (20)	-
MM (30)	-
PDA	7.8
YM	15.6
MEA	62.5
Corn meal	15.6
Itraconazole	0.001

MEA: malt extract agar; MM: minimal medium supplemented with 5-30 g/L glucose; PDA: potato dextrose agar; YM: yeast mold; -: no activity.

As the ethanolic extract of *A. felis* UFMGCB 8030 grown on PDA demonstrated the lowest MIC, this medium was used to identify the optimal solvent for the production of extracts with the highest antifungal activity. The extract obtained using DCM was found to be the most active against *P. brasiliensisPb*18 (MIC = 1.9 µg/mL), followed by that produced with ethanol (MIC = 7.8 µg/mL). Extracts prepared with ethyl acetate and hexane were only minimally active (with MICs of 500.0 and 250.0 µg/mL, respectively) (Table III). The PDA/DCM *A. felis* UFMGCB 8030 extract did not show cytotoxicity at the concentrations tested when assayed with Vero cells, demonstrating that this extract exhibits some selectivity towards fungal cells compared to mammalian cells.

**TABLE III:** Minimum inhibitory concentrations (MIC) against*Paracoccidioides brasiliensisPb*18 of various solvent extracts from *Aspergillus felis* (UFMGCB 8030) cultures grown on potato dextrose agar

Solvent	MIC (µg/mL)
Hexane	250.0
Dichloromethane	1.9
Ethyl acetate	500.0
Ethanol	7.8

**Fig. 3:**
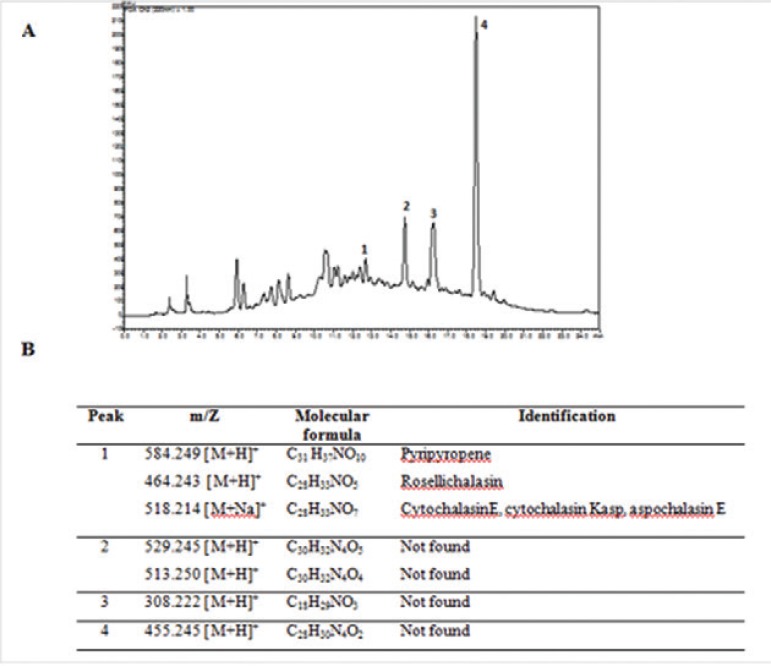
: identification of secondary metabolites in dichloromethane (DCM) extract of *Aspergillus felis* UFMGCB 8030 grown for 15 days on potato dextrose agar medium. A: high-performance liquid chromatography chromatogram of *A. felis* DCM extract (ultraviolet detection at 220 nm) showing active fractions 1-4 (≥ 70% inhibition of isolate *Pb*18 growth); B: table showing the base-peak values of active fractions 1-4 with their molecular formulae and manual verification of high resolution mass spectrometry results using SciFinder and KNApSAcK data.

The PDA/DCM extract was then subjected to bioassay-guided fractionation using RP-HPLC and a *P. brasi- liensis* assay (Fig. 3). The active fractions were analysed by HPLC-high resolution mass spectrometry (HRMS) with electrospray ionisation in positive-ion mode to obtain accurate mass measurements. A tentative identification based on the resulting mass spectra was achieved by manual verification using SciFinder and KNApSAcK data. The HRMS data corresponding to active fraction 1 consisted of *m/z*signals at 584.249 [M+H]^+^, 464.243 [M+H]^+^, and 518.214 [M+Na]^+^ that were tentatively identified as known compounds pyripyropene A (Omura et al. 1993), rosellichalasin (Kimura et al. 1989), and cytochalasin E (Aldridge et al. 1972), cytochalasin Kasp (Kimura et al. 1989), or aspochalasin E (Steyn et al. 1982), respectively. Active fraction 1 comprised multiple compounds, but the effective identification of these based on patterns of substitution was not possible due to a lack of information in the literature. The resulting formulas obtained from the fractions 2-4 did not match against SciFinder and KNApSAcK database to search for known metabolites. It could be hypothesised that these fractions can contain metabolites that were not previously isolated from *Aspergillus*species.

## DISCUSSION

In the present work, the DCM extract of *A. felis* UFMGCB 8030 displayed promising activity against *P. brasiliensis Pb*18, although in a previous screen with *Candida albicans*, *Candida krusei*, and *Cladosporium sphaerospermum* it was shown to be inactive (Gonçalves et al. 2015). Although this fungus has previously been identified using ITS sequences (Gonçalves et al. 2015), in this work the identity of isolate UFMGCB 8030 was confirmed using molecular, morphological, and phylogenetic methodologies. According to Barrs et al. (2013), species belonging to the *Aspergillus*, section*Fumigati*, cannot be identified only on the basis of morphological aspects only, therefore the use of other approaches for the identification of such organisms is key. Barrs et al. (2013) recently described the identification of *A. felis*in human and animal hosts (dogs and cats) with invasive aspergillosis. The isolation of this fungus from environmental samples was first reported by our group, as a result of an investigation of Atacama Desert’s rock samples (Gonçalves et al. 2015).

In fungi, the biosynthesis of secondary metabolites is regulated in response to nutrient availability or as a result of changes in the environment or developmental phase (Sanchez & Demain 2002, Zain et al. 2011). Altering the media used to culture microorganisms can enhance the production of bioactive compounds (Abdel-Fattah & Olama 2002). A good understanding of the role of culture conditions in the biosynthesis of metabolites may lead to improved exploitation of microorganisms-derived compounds (Miao et al. 2006). The ethanol extract obtained by cultivation of *A. felis*UFMGCB 8030 on PDA resulted in the strongest effect, i.e., the lowest MIC, in an evaluation of culture media, while minimal medium failed to provide conditions suitable for the production of antifungal compounds against *Pb*18.Bhattacharyya and Jha (2011) showed that in salt-rich media such as Czapek-Dox, the growth and antimicrobial activity of an*Aspergillus* strain was lower than that observed using a complex medium such as PDA. In addition, Mathan et al. (2013) demonstrated that low-nutrient medium has a detrimental effect on mycelial growth and metabolite profile in *Aspergillus terreus*. This suggests that in salt-rich or nutrient-poor media, mycelial growth interferes with the production of antifungal metabolites by *Aspergillus* spp.

As *A. felis* was described only very recently, we were unable to find any records in the literature concerning investigation of its secondary metabolites, a fact that encouraged us to determine the compounds in the UFMGCB 8030 DCM extract responsible for its antifungal activity.

Concerning the compounds identified in this extract, no reports of antifungal activity exist for pyripyropene, rosellichalasin, cytochalasin Kasp, or aspochalasin E. However, cytochalasin E has been tested against *Fusarium solani*(MIC > 100 µM), *Gibberella saubinetti* (MIC = 100 µM),*Botrytis cinerea* (MIC = 100 µM), and *Alternaria solani* (MIC = 50 µM), showing weak antifungal activity with MIC values generally greater than 50 µM (Zhang et al. 2014). Although antifungal activity against organisms of agricultural importance has thus been documented, no investigations into the effect of the compounds identified in this work against fungi of medical interest have been carried out.

The fast tentative identification of natural products using the dereplication process can be very efficient to detect promising source of new bioactive compounds (Kildgaard et al. 2014, Petersen et al. 2014, Boruta & Bizukojc 2015). In one of the fractions displaying antifungal activity, cytochalasins were identified as the active metabolites. Cytochalasins are a group of fungal secondary metabolites with a 10-phenylperhydroisoindol-1-one skeleton and a macrocyclic ring and are capable of various biological activities (Qiao et al. 2011). They have been described not only in the genus*Aspergillus* (Demain et al. 1976, Udagawa et al. 2000, Lin et al. 2009, Zheng et al. 2013), but also in *Xylaria* (Silva et al. 2010),*Cladosporium* (Cafêu et al. 2005), *Arthrinium* (Wang 2015), and*Phomopsis* (Shen et al. 2014). According to Guerra et al. (2014), cytochalasins inhibit actin polimerisation and act preventing actin interaction with host cells in the fungal pathogen *Cryptococcus neoformans.C. neoformans* is internalised by receptor-mediated or “triggered” phagocytosis, dependent on actin recruitment. Additionally, they can act as microfilament-disrupting agents, alter cell motility, adherence, secretion, drug efflux, deformability, morphology, and size, among many other cell properties critical to neoplastic cell pathology (Van Goietsenoven et al. 2011). Rosellichalasin and cytochalasin E isolated from *Aspergillus* sp. exhibit potent cytotoxic activity against human tumour cell lines (Xiao et al. 2013). Besides these compounds, aspochalasin E shows potent activity against murine melanoma B16-F10 and human colon carcinoma HCT-116 cells (Naruse et al. 1993). Pyripyropene A acts in decrease of intestinal cholesterol absorption and cholesteryl oleate levels, resulting in protection of atherosclerosis development (Ohshiro et al. 2011).

The literature contains few reports on the isolation of compounds from fungi exhibiting activity against *P. brasiliensis*. However, among these are altenusin, isolated from an *Alternaria* sp. (Johann et al. 2012), and trichothecene mycotoxins (T-2 toxin and a mixture of 8-*n*-isobutyrylsolaniol and 8-*n*-butyryl*neo*solaniol (Campos et al. 2011).

This study indicated that fungi isolated from Atacama Desert rocks may constitute potential sources of novel bioactive compounds. *A. felis* UFMGCB 8030 produced the most active extract among those studied and its antifungal activity was enhanced by changes in culture conditions. The DCM extract of this fungus showed low cytotoxicity in preliminary tests and outstanding activity against one of the fungi responsible for PCM. Our results demonstrate the importance of further studies into the fungus *A. felis*, since the analyses presented here suggest that previously unknown bioactive compounds can be produced by this species.

## References

[B1] Abadio AKR, Kioshima ES, Leroux V, Martins NF, Maigret B, Felipe MSS (2015). Identification of new antifungal compounds targeting thioredoxin
redutase of Paracoccidioides genus. Plos ONE.

[B2] Abdel-Fattah YR, Olama ZA (2002). L-asparaginase production by Pseudomonas aeruginosa in solid-state
culture: evaluation and optimization of culture conditions using factorial
designs. Process Biochem.

[B3] Aldridge DC, Burrows BF, Turner WB (1972). The structures of the fungal metabolites cytochalasins E and
F. J Chem Soc Chem Commun.

[B4] Altschul SF, Madden TL, Schaffer AA, Zhang JH, Zhang Z, Miller W, Lipman DJ (1997). Gapped BLAST and PSI-BLAST: a new generation of protein database
search programs. Nucleic Acids Res.

[B5] Andrade RV, Silva SP, Torres FAG, Poças-Fonseca MJ, Silva-Pereira I, Maranhão AQ, Campos EG, Moraes LMP, Jesuíno RSA, Pereira M, Soares CMA, Walter ME, Carvalho MJA, Almeida NF, Brígido MM, Felipe MSS (2005). Overview and perspectives on the transcriptome of^Paracoccidioides
brasiliensis^. Rev Iberoam Micol.

[B6] Araújo FS, Coelho LM, Silva LC, Silva BR, Parente-Rocha JA, Bailão AM, Oliveira CM, Fernandes GR, Hérnandez O, Ochoa JG, Soares CM, Pereira M (2016). Effects of argentillactone on the transcriptional profile, cell wall,
and oxidative stress of Paracoccidioides spp.. Plos Negl Trop Dis.

[B7] Azua-Bustos A, Urrejola C, Vicuña R (2012). Life at the dry edge: microorganisms of the Atacama
Desert. Febs Letters.

[B8] Barrs VR, Tineke MD, Houbraken J, Kidd SE, Martin P, Pinheiro MD, Richardson M, Varga J, Samson RA (2013). Aspergillus felis sp. nov., an emerging agent of invasive
Aspergillosis in humans, cats, and dogs. PLoS ONE.

[B9] Bhattacharyya PN, Jha DK (2011). Optimization of cultural conditions affecting growth and improved
bioactive metabolite production by a subsurface Aspergillus strain tsf
146. Int J Appl Biol Pharm.

[B10] Borges-Walmsley MI, Chen D, Shu X, Walmsley AR (2002). The pathobiology of Paracoccidioides brasiliensis. Trends Microbiol.

[B11] Boruta T, Bizukojc M (2015). Induction of secondary metabolism of Aspergillus terreus ATCC 20542 in
the batch bioreactor cultures. Appl Microbiol Biotechnol.

[B12] Cafêu MC, Silva GH, Teles HL, Bolzani VS, Araújo AR, Young MCM, Pfenning LH (2005). Antifungal compounds of Xylaria sp., an endophytic fungus isolated
from Palicourea marcgravii (Rubiaceae). Quim Nova.

[B13] Campos FF, Johann S, Cota BB, Alves TM, Rosa LH, Caligiorne RB, Cisalpino OS, Rosa CA, Zani CL (2011). Antifungal activity of trichothecenes from Fusarium sp. against
clinical isolates of Paracoccidioides brasiliensis. Mycoses.

[B14] CLSI - Clinical Laboratory Standards Institute (2008). Reference method for broth dilution antifungal susceptibility testing of
yeasts, CLSI document M27-A3, Approved standard.

[B15] Cruz RC, Werneck SMC, Oliveira CS, Santos PC, Soares BM, Santos DA, Cisalpino PS (2012). Conditions for determining the minimal inhibitory concentration (MIC)
of seven antifungal agents against Paracoccidioides brasiliensis by microdilution:
influence of different media, incubation times, and temperatures. J Clin Microbiol.

[B16] Dalmaso GZL, Ferreira D, Vermelho AB (2015). Marine extremophiles: a source of hydrolases for biotechnological
applications. Mar Drugs.

[B17] Demain AL, Hunt NA, Malik V, Kobbe B, Hawkins H, Matsuo K, Wogan GN (1976). Improved procedure for production of cytochalasin E and tremorgenic
mycotoxins by Aspergillus clavatus. Appl Environ Microbiol.

[B18] Glass NL, Donaldson GC (1995). Development of primer sets designed for use with the PCR to amplify
conserved genes from filamentous ascomycetes. Appl Environ Microbiol.

[B19] Godinho VM, Furbino LE, Santiago IF, Pellizzari FM, Yokoya N, Pupo D, Alves TMA, Júnior PAS, Romanha AJ, Zani CL, Cantrell CL, Rosa CA, Rosa LH (2013). Diversity and bioprospecting of fungal communities associated with
endemic and cold-adapted macroalgae in Antarctica. ISME J.

[B20] Gonçalves VN, Cantrell CL, Wedge DE, Ferreira MC, Soares MA, Jacob MR, Oliveira FS, Galante D, Rodrigues F, Alves TMA, Zani CL, Júnior PAS, Murta S, Romanha AJ, Barbosa EC, Kroon EG, Oliveira JG, Gómez-Silva B, Galetovic A, Rosa CA, Rosa LH (2015). Fungi associated with rocks of the Atacama Desert: taxonomy,
distribution, diversity, ecology, and bioprospection for bioactive
compounds. Environ Microbiol.

[B21] Gueidan C, Villaseñor CR, Hoog GS, Gorbushina AA, Untereiner WA, Lutzoni F (2008). A rock-inhabiting ancestor for mutualistic and pathogen-rich fungal
lineages. Stud Mycol.

[B22] Guerra CR, Seabra SH, Souza W, Rozental S (2014). Cryptococcus neoformans is internalized by receptor-mediated or
“triggered” phagocytosis, dependent on actin recruitment. PLoS ONE.

[B23] Horai H, Arita M, Kanaya S, Nihei Y, Ikeda T, Suwa K, Ojima Y, Tanaka K, Tanaka S, Aoshima K, Oda Y, Kakazu Y, Kusano M, Tohge T, Matsuda F, Sawada Y, Hirai MY, Nakanishi H, Ikeda K, Akimoto N, Maoka T, Takahashi H, Ara T, Sakurai N, Suzuki H, Shibata D, Neumann S, Iida T, Tanaka K, Funatsu K, Matsuura F, Soga T, Taguchi R, Saito K, Nishioka T (2010). MassBank: a public repository for sharing mass spectral data for life
sciences. J Mass Spectrom.

[B24] Houbraken J, Frisvad JC, Seifert KA, Overy DP, Tuthill DM, Valdez JG, Samson RA (2012). New penicillin-producing Penicillium species and an overview of
section Chrysogena. Persoonia.

[B25] Johann S, Cisalpino OS, Watanabe GA, Cota BB, Siqueira EP, Pizzolati MG, Zani CL, Resende MA (2010). Antifungal activity of extracts of some plants used in the Brazilian
traditional medicine against the pathogenic fungus^Paracoccidioides
brasiliensis^. Pharm Biol.

[B26] Johann S, Rosa LH, Rosa CA, Perez P, Cisalpino PS, Zani CL, Cota BC (2012). Antifungal activity of altenusin isolated from the endophytic fungus
Alternaria sp. against the pathogenic fungus Paracoccidioides
brasiliensis. Rev Iberoam Micol.

[B27] Kildgaard S, Mansson M, Dosen I, Klitgaard A, Frisvad JC, Larsen TO, Nielsen KF (2014). Accurate dereplication of bioactive secondary metabolites from
marine-derived fungi by UHPLC-DAD-QTOFMS and a MS/HRMS library. Mar Drugs.

[B28] Kimura Y, Nakajima H, Hamasaki T (1989). Structure of rosellichalasin, a new metabolite produced by Rosellinia
necatrix. Agric Biol Chem.

[B29] Kirk PM, Cannon PF, Minter DW, Stalpers JA (2008). Dictionary of the fungi.

[B30] Klich MA (2002). Identification of common Aspergillus species.

[B31] Lin Z, Zhang G, Zhu T, Liu R, Wei H, Gu Q (2009). Bioactive cytochalasins from Aspergillus flavipes, an endophytic
fungus associated with the mangrove plant Acanthus ilicifolius. Helv Chim Acta.

[B32] Mathan S, Subramanian V, Nagamony S (2013). Optimization and antimicrobial metabolite production from endophytic
fungi Aspergillus terreus KC 582297. Euro J Exp Bio.

[B33] Matute DR, McEwen JG, Puccia R, Montes BA, San-Blas G, Bagagli E, Rauscher JT, Restrepo A, Morais F, Niño-Vega G, Taylor JW (2006). Cryptic speciation and recombination in the fungus Paracoccidioides
brasiliensis as revealed by gene genealogies. Mol Biol Evol.

[B34] Li Miao, Kwong TFN, Qian P (2006). Effect of culture conditions on mycelial growth, antibacterial
activity, and metabolite profiles of the marine-derived fungus Arthrinium c.f.
saccharicola.. Appl Microbiol Biotechnol.

[B35] Monks A, Scudiero D, Skehan P, Shoemaker R, Paull K, Vistica D, Hose C, Langley J, Cronise P, Vaigro-Wolff A (1991). Feasibility of a high-flux anticancer drug screen using a diverse
panel of cultured human tumor cell lines. J Natl Cancer Inst.

[B36] Naruse N, Yamamoto H, Murata S, Sawsa Y, Fukagawa Y, Oki T (1993). Aspochalasin E, a new antibiotic isolated from a
fungus. J Antibiot (Tokyo).

[B37] Ohshiro T, Matsuda D, Sakai K, Degirolamo C, Yagyu H, Rudel LL, Omura S, Ishibashi S, Tomoda H (2011). Pyripyropene A, an acyl-coenzyme A: cholesterol acyltransferase
2-selective inhibitor, attenuates hypercholesterolemia and atherosclerosis in
murine models of hyperlipidemia. Arterioscler Thromb Vasc Biol.

[B38] Omura S, Tomoda H, Kim YK, Nishida H (1993). Pyripyropenes, high potent inhibitors of acyl-CoA cholesterol
acyltransferase produced by Aspergillus fumigates. J Antibiot (Tokyo).

[B39] Palmeiro M, Cherubini K, Yurgel LS (2005). Paracoccidioidomicose - Revisão da literatura. Sci Med.

[B40] Petersen LM, Hoeck C, Frisvad JC, Gotfredsen CH, Larsen TO (2014). Dereplication guided discovery of secondary metabolites of mixed
biosynthetic origin from Aspergillus aculeatus. Molecules.

[B41] Prado RS, Bailão AM, Silva LC, Oliveira CMA, Marques MF, Silva LP, Silveira-Lacerda EP, Lima AP, Soares CM, Pereira M (2015). Proteomic profile response of Paracoccidioides lutzii to the
antifungal argentilactone. Front Microbiol.

[B42] k Qiao, Chooi YH, Tang Y (2011). Identification and engineering of the cytochalasin gene cluster from
Aspergillus clavatus NRRL 1. Metab Eng.

[B43] Rosa LH, Queiroz SCN, Moraes RM, Wang X, Techen N, Pan Z, Charles L, Cantrell CL, Wedge DE (2013). Coniochaeta ligniaria: antifungal activity of the cryptic endophytic
fungus associated with autotrophic tissue cultures of the medicinal plant
Smallanthus sonchifolius (Asteraceae). Symbiosis.

[B44] Rosa LH, Vaz ABM, Caligiorne RB, Campolina S, Rosa CA (2009). Endophytic fungi associated with the Antartic grass Deschampsia
antarctica Desv. (Poaceae). Polar Biol.

[B45] Sanchez S, Demain AL (2002). Regulation of fermentation processes. Enzyme Microb Technol.

[B46] l Shen, Luo Q, Shen Z, Li L, Zhang X, Wei Z, Fu Y, Tan R, Song Y (2014). A new cytochalasin from endophytic Phomopsis sp.
IFB-E060. Chin J Nat Med.

[B47] Shikanai-Yasuda MA, Telles FQ, Mendes RP, Colombo AI, Moretti MI (2006). Guidelines in paracoccidioidomycosis. Rev Soc Bras Med Trop.

[B48] Silva GH, Oliveira CM, Teles HL, Bolzani VS, Araújo AR, Pfenning LH, Young MCM, Costa CM, Haddad R, Eberlin MN (2010). Citocalasinas produzidas por Xylaria sp., um fungo endofítico de Piper
aduncum (Piperaceae). Quim Nova.

[B49] Steyn PS, van Heerden FR, Rabie CJ (1982). Cytochalasins E and K, toxic metabolites from Aspergillus
clavatus. J Chem Soc.

[B50] Stürme MHJ, Puccia R, Goldman GH, Rodrigues F (2011). Molecular biology of the dimorphic fungi Paracoccidioides
spp.. Fungal Biol Rev.

[B51] Tamura K, Peterson D, Peterson N, Stecher G, Nei M, Kumar S (2011). MEGA5: molecular evolutionary genetics analysis using maximum
likelihood, evolutionary distance, and maximum parsimony methods. Mol Biol Evol.

[B52] Tavares AH, Silva SS, Bernardes VV, Maranhão AQ, Kyaw CM, Poças-Fonseca M, Silva-Pereira I (2005). Virulence insights from the Paracoccidioides brasiliensis
transcriptome. Genet Mol Res.

[B53] Teixeira MM, Theodoro RC, Carvalho MJA, Fernandes L, Paes HC, Hahn RC, Mendoza L, Bagagli E, San-Blas G, Felipe MS (2009). Phylogenetic analysis reveals a high level of speciation in the
Paracoccidioides genus. Mol Phylogenet Evol.

[B54] Tesei D, Marzban G, Zakharova K, Isola D, Selbmann L, Sterflinger K (2012). Alteration of protein patterns in black rock inhabiting fungi as a
response to different temperatures. Fungal Biol.

[B55] Torres I, Garcia AM, Hernández O, González A, McEwen JG, Restrepo A, Arango M (2010). Presence and expression of the mating type locus in Paracoccidioides
brasiliensis isolates. Fungal Genet Biol.

[B56] Travassos LR, Taborda CP (2012). New advances in the development of a vaccine against
paracoccidioidomycosis. Front Microbiol.

[B57] Udagawa T, Yuan J, Panigrahy D, Chang YH, Shah J, D’Amato RJ (2000). Cytochalasin E, an epoxide containing Aspergillus-derived fungal
metabolite, inhibits angiogenesis and tumor growth. J Pharmacol Exp Ther.

[B58] Van Goietsenoven G, Mathieu V, Andolfi A, Cimmino A, Lefranc F, Kiss R, Evidente A (2011). In vitro growth inhibitory effects of cytochalasins and derivatives in
cancer cells. Planta Med.

[B59] Visbal G, San-Blas G, Maldonado A, Alvarez-Aular A, Capparelli MV, Murgich J (2011). Synthesis, in vitro antifungal activity and mechanism of action of
four sterol hydrazone analogues against the dimorphic fungus Paracoccidioides
brasiliensis.. Steroids.

[B60] Wang J, Wang Z, Ju Z, Wan J, Liao S, Lin X, Zhang T, Zhou X, Chen H, Tu Z, Liu Y (2015). Cytotoxic cytochalasins from marine-derived fungus Arthrinium
arundinis. Planta Med.

[B61] White TJ, Bruns TD, Lee SB, Taylor J, Innis MA, Gelfand DH, Shinsky JJ, White TJ (1990). Amplification and direct sequencing of fungal ribosomal
RNA genes for phylogenetics. PCR protocols: a guide to methods and applications.

[B62] Wierzchos J, Davila AF, Artieda O, Cámara-Gallego B, Los Ríos A, Nealson KH, Valea S, García-González MT, Ascaso C (2013). Ignimbrite as a substrate for endolithic life in the hyper-arid
Atacama Desert: implications for the search for life on Mars. Icarus.

[B63] Xiao L, Liu H, Wu N, Liu M, Wei J, Zhang Y, Lin X (2013). Characterization of the high cytochalasin E and rosellichalasin
producing-Aspergillus sp. nov. F1 isolated from marine solar saltern in
China. World J Microbiol Biotechnol.

[B64] Zain ME, Razak AA, El-Sheikh HH, Soliman HG, Khalil AM (2011). Influence of growth medium on diagnostic characters of Aspergillus and
Penicillium species. Afr J Microbiol Res.

[B65] Zhang Q, Xiao J, Sun Q, Qin J, Pescitelli G, Gao J (2014). Characterization of cytochalasins from the endophytic Xylaria sp. and
their biological functions. J Agric Food Chem.

[B66] Zheng C, Shao C, Lu Wu, Chen M, Wang K, Zhao D, Sun X, Chen G, Wang C (2013). Bioactive phenylalanine derivatives and cytochalasins from the soft
coral-derived fungus, Aspergillus elegans. Mar Drugs.

